# The future of peer review: an editorial

**DOI:** 10.3399/BJGPO.2024.0285

**Published:** 2024-12-11

**Authors:** Adam Grice, Hajira Dambha-Miller

**Affiliations:** 1 BJGP Open, London, UK

**Keywords:** general practice, editorial, peer review, primary healthcare

As *BJGP Open* recently received its first impact factor, and the number of submissions this year reached an all-time high, we are reviewing our editorial processes and looking to the future of peer review and publishing. With the increasing volume of submissions, there is also a growing demand and expectation from authors for rapid decisions and quicker time to publication. Balancing these expectations with the need to maintain rigorous quality standards presents a significant challenge. Academic journals play a critical role in advancing scientific knowledge and ensuring that research contributes meaningfully to the progress of a field. A key responsibility of journals is to publish high quality studies that provide valuable insights while actively working to reduce research waste and unnecessary duplication. Publishing research for the sake of publishing is a common problem. It not only clutters the scientific literature but diverts attention from studies that could truly advance understanding and improve practice. By carefully selecting manuscripts for publication, we help ensure that only those studies that meet rigorous standards of quality, methodology, and relevance are disseminated.

Reducing research waste is a major challenge facing the scientific community.^1^ It includes addressing issues such as poor study design, publication bias, and a lack of methodological rigour, which can lead to conclusions that do not advance knowledge or improve outcomes. As editors, we act as gatekeepers, prioritising studies that are methodologically sound, well designed, and likely to lead to actionable results for primary care practitioners. This role has become even more critical as the volume of research continues to increase, with many studies failing to add value or simply replicating existing findings. By enforcing robust peer review and providing constructive feedback, we can help researchers refine their work, ensuring it is of the highest quality before publication.

Minimising duplication of research includes ensuring that studies build upon existing knowledge rather than repeating experiments that have already been conducted. The peer review process plays an important role in this regard, as it helps identify previous work that may be similar or identical to new submissions. We also facilitate the sharing of research data and findings through open access publishing, which helps to reduce duplication and encourages further collaboration across research teams and disciplines. This not only optimises resources but accelerates scientific progress by ensuring that research findings are widely accessible and can be built upon by others.

The peer review process itself has evolved significantly in recent years. One major change that we have adopted is the move towards group manuscript meetings, where multiple reviewers and members of the editorial board discuss and deliberate on the merits of an article before making a collective decision. This collaborative approach ensures that multiple perspectives are considered, which can lead to more comprehensive and balanced evaluations. These meetings also allow for a more nuanced discussion of articles, enabling us to make decisions based on a broader array of expert opinions. This collective decision-making process ensures greater transparency and accountability in the publication process, which can ultimately improve the quality of published research.

In addition to these changes, we have seen a greater involvement of associate editors in the peer review process. Associate editors now play a critical role in managing the review process, providing detailed evaluations, and offering more direct feedback to authors. This expanded role helps ease the workload on senior editors, ensuring that reviews are thorough and timely while also increasing the expertise applied to each submission. By involving a broader team of experts in the review process, we ensure that each manuscript receives the attention it deserves and is evaluated with a more diverse range of insights.

Our editorial fellowship program has also proven to be an invaluable part of our approach to developing future leaders in scientific publishing. These fellowships offer early-career colleagues an excellent opportunity to engage in the editorial process, providing them with great learning experiences. Fellows are exposed to every step of the editorial workflow, from handling submissions to participating in peer reviews and editorial discussions. This mentorship not only enhances the skills of emerging researchers, but also supports the journal in handling the increasing number of submissions while fostering a deeper understanding of the scientific publishing process. These fellowships serve as an important developmental tool, helping to build a new generation of experts who can contribute to advancing knowledge in meaningful ways.

However, challenges around peer review are forcing us to approach this process differently now. The increase in electronic journals, and therefore the increase in submissions, as well as the impact of the COVID-19 pandemic, has contributed to shortages of reviewers, which subsequently delays decisions and possibly impacts the quality of reviews.^2^ With the rising volume of submissions, many journals are facing difficulties in securing qualified reviewers in a timely manner. This backlog, combined with increasing pressures on the research community, has made it evident that we need to rethink how peer review is structured and carried out to maintain the quality and integrity of the publication process.

Looking ahead, we are growing our senior editorial staff and increasingly considering the role of artificial intelligence (AI) to help transform the peer review process for the better. When built into the submission and peer review systems, AI tools have the potential to significantly enhance the efficiency, accuracy, and quality of peer review.^3^ These tools may assist in a variety of ways, improving the overall review process. AI can automatically detect methodological flaws, inconsistencies in data, or issues with clarity in argumentation that might otherwise be overlooked by human reviewers. For example, AI can quickly spot statistical errors or misinterpretations of data that are often time-consuming for humans to identify. Additionally, AI systems can evaluate the completeness of a manuscript by checking the structure, ensuring that important sections such as the methodology, results, and discussion are sufficiently detailed.

Beyond simply identifying flaws, AI can also help with the logistics of peer review by matching manuscripts to the most appropriate reviewers. By analysing reviewers' past publications, expertise, and feedback history, AI can recommend individuals who are best suited to evaluate specific aspects of an article. This streamlines the process of assigning manuscripts and ensures that the most qualified reviewers are chosen, even in cases where there may be a shortage of available experts.

AI tools can also assist with tasks like detecting plagiarism,^4^ ensuring compliance with ethical guidelines, and identifying potential conflicts of interest, all of which can help safeguard the integrity of the review process. Furthermore, AI can provide real-time feedback to authors, suggesting improvements or highlighting issues that need addressing, which can ultimately speed up the revision process and improve the quality of the final manuscript.

While AI will never replace the nuanced judgment and expertise of human reviewers,^5^ it serves as a powerful supplement that can potentially streamline workflows, improve efficiency, and enhance the overall quality of reviews. In the future, we expect that AI could play an even larger role in the peer review process, automating routine tasks, assisting in decision-making, and providing authors and reviewers with valuable insights. However, the human element remains indispensable in assessing the broader significance, context, and potential impact of the research.

As the landscape of research dissemination continues to evolve, with the shift to open access publishing, we as a journal must adapt to meet the changing needs of the scientific community. Open access has the potential to democratise access to research, but it also places additional pressure on journals to maintain sustainability and ensure the integrity of the studies we publish.^6^ Despite these challenges, the peer review process remains essential for maintaining high standards, as it provides an opportunity for expert scrutiny, improving the overall quality of published work. In scientific publishing, we must continue to prioritise the advancement of knowledge while reducing research waste and avoiding duplication. By evolving the peer review process — expanding the role of associate editors, integrating AI tools, exploring new ways to manage reviewer shortages, and fostering opportunities for early career staff through editorial fellowships — we can enhance the quality and efficiency of reviews. These changes ensure that we publish high quality, impactful research, safeguarding the integrity of the scientific process and advancing progress in our field.

## Data

N/A
